# Anti-Cytosolic 5′-Nucleotidase 1A in the Diagnosis of Patients with Suspected Idiopathic Inflammatory Myopathies: An Italian Real-Life, Single-Centre Retrospective Study

**DOI:** 10.3390/biomedicines11071963

**Published:** 2023-07-12

**Authors:** Brunetta Porcelli, Miriana d’Alessandro, Latika Gupta, Silvia Grazzini, Nila Volpi, Maria Romana Bacarelli, Federica Ginanneschi, Giovanni Biasi, Francesca Bellisai, Marta Fabbroni, David Bennett, Claudia Fabiani, Luca Cantarini, Elena Bargagli, Bruno Frediani, Edoardo Conticini

**Affiliations:** 1 UOC Laboratorio Patologia Clinica, Policlinico S. Maria alle Scotte, AOU Senese, 53100 Siena, Italy; 2 Dipartimento Biotecnologie Mediche, Università degli Studi di Siena, 53100 Siena, Italy; 3 Respiratory Diseases Unit, Department of Medical and Surgical Sciences & Neurosciences, University of Siena, 53100 Siena, Italy; 4 Department of Clinical Immunology and Rheumatology, Sanjay Gandhi Postgraduate Institute of Medical Sciences, Lucknow 226014, India; 5 Rheumatology Unit, Department of Medicine, Surgery and Neurosciences, University of Siena, 53100 Siena, Italy; 6 Neurology and Clinical Neurophysiology Unit, Department of Medical, Surgical and Neurological Sciences, University of Siena, 53100 Siena, Italy; 7 Ophthalmology Unit, Department of Medicine, Surgery and Neurosciences, University of Siena, 53100 Siena, Italy

**Keywords:** idiopathic inflammatory myopathies, autoantibodies, inclusion body myositis

## Abstract

Background: Anti-cytosolic 5′-nucleotidase 1A (anti-cN1A) antibodies were proposed as a biomarker for the diagnosis of inclusion body myositis (IBM), but conflicting specificity and sensitivity evidence limits its use. Our study aimed to assess the diagnostic accuracy of anti-cN1A in a cohort of patients who underwent a myositis line immunoassay for suspected idiopathic inflammatory myopathies (IIM). We also assessed the agreement between two testing procedures: line immunoassay (LIA) and enzyme-linked immunoassay (ELISA). Materials and methods: We collected retrospective clinical and serological data for 340 patients who underwent a myositis antibody assay using LIA (EUROLINE Autoimmune Inflammatory Myopathies 16 Ag et cN-1A (IgG) line immunoassay) and verification with an anti-cN1A antibody assay using ELISA (IgG) (Euroimmun Lubeck, Germany). Results: The serum samples of 20 (5.88%) patients (15 females, 5 males, mean age 58.76 ± 18.31) tested positive for anti-cN1A using LIA, but only two out of twenty were diagnosed with IBM. Seventeen out of twenty tested positive for anti-cN1A using ELISA (median IQR, 2.9 (1.9–4.18)). Conclusions: Our study suggests excellent concordance between LIA and ELISA for detecting anti-cN1A antibodies. LIA may be a rapid and useful adjunct, and it could even replace ELISA for cN1A assay. However, the high prevalence of diseases other than IBM in our cohort of anti-cN1A-positive patients did not allow us to consider anti-cN1A antibodies as a specific biomarker for IBM.

## 1. Introduction

Inclusion body myositis (IBM) is a rare progressive autoimmune myopathy with an overall prevalence of 84 per million, typically affecting persons over 50 years of age [1]. It is more common in men and is characterised by the typical involvement of the finger flexors, ankle dorsiflexors and knee extensors. Although it is considered as a degenerative disorder with few therapeutic options and poor, if any, response to conventional immunosuppressants, recent findings suggest that the immune system may play a role in its development [2,3]. This is important because it opens a window of opportunity for targeted treatments.

Anti-cytosolic 5′-nucleotidase 1A (anti-cN1A) antibodies were proposed as a diagnostic biomarker for IBM by virtue of their high specificity (87–100%) [4,5]. Nevertheless, their use in the diagnostic work-up of IBM is still limited by the controversial evidence of sensitivity (33–76%) and the limited availability of tests for these antibodies in most clinical laboratories [6].

Little and controversial data suggest that anti-cN1A could be a marker of prognosis and a response to treatment in patients with a definite diagnosis of IBM. Lucchini et al. suggested a higher prevalence of dysphagia in anti-cN1A-positive subjects [4], while other authors [7,8] played down the clinical significance of these antibodies. There are also unanswered questions about anti-cN1A positivity in other autoimmune disorders and its association with specific clinical features in non-IBM patients.

Standardised tests for anti-cN1A are still lacking [8]. Anti-cN1A analysis was first performed with western blot and immunoprecipitation [9,10]. In 2016, an enzyme-linked immunoassay (ELISA) with a higher specificity and sensitivity was developed [8,11]. In the context of the expanding spectrum of myositis-specific autoantibodies (MSA), a LIA for MSA—including anti-cN1A that could save time, materials and labour costs—was recently developed. This test enabled a fast, simple assessment of several antibodies simultaneously.

The aim of this study was to assess the diagnostic accuracy of anti-cN1A in a cohort of Italian patients who underwent the analysis of myositis antibodies with LIA for suspected idiopathic inflammatory myopathies (IIM). Our second aim was to assess the agreement between LIA and ELISA testing procedures.

## 2. Materials and Methods

### 2.1. Study Population

We collected retrospective clinical and serological data of all patients who underwent myositis antibody analysis with LIA at the University Hospital of Siena, Italy, from August 2020 to December 2021. Exclusion criteria were a previous diagnosis of IBM, or any autoimmune rheumatic disease, or the lack of clinical and laboratory data. The STROBE checklist [12] was used for the Section 2.

### 2.2. Diagnostic Criteria

Patients were diagnosed with definite or probable IBM when they fulfilled 2011 ENMC research diagnostic criteria [13]. Dermatomyositis and polymyositis were defined according to ACR/EULAR criteria (definite or probable). Overlap myositis was diagnosed in patients who fulfilled both the Bohan–Peter [14] and any one criterion for connective tissue disease. Anti-synthetase syndrome was defined according to Lega classification criteria [15]. Rheumatoid arthritis and spondylarthritis were diagnosed according to ACR/EULAR and ASAS criteria. Systemic lupus erythematosus, systemic sclerosis and Sjögren’s syndrome were defined according to EULAR/ACR classification criteria [16,17,18].

### 2.3. Clinical Details

For each patient who tested positive for anti-cN1A antibodies, the following data were recorded in an electronic database: age, sex, definite diagnosis, date of onset of symptoms, date of diagnosis, risk factors, clinical features (including dysphagia, ILD, heart involvement, gastrointestinal involvement, arthralgia/arthritis, muscle weakness, skin involvement), muscle biopsy, magnetic resonance imaging and electromyography (EMG) findings (when available), other autoantibodies tested in common clinical practice and outcome. Clinical data were collected by two rheumatologists and a pneumologist, all with expertise in IIM.

### 2.4. cN1A Assay

Anti-cN1A antibody was detected in all 340 patients using EUROLINE Autoimmune Inflammatory Myopathies 16 Ag et cN-1A (IgG) (Euroimmun, Lubeck, Germany) [19], a multiparameter LIA which provides qualitative determination of immunoglobulin class IgG autoantibodies to 17 different antigens: Mi-2α, Mi-2β, TIF1γ, MDA5, NXP2, SAE1, Ku, PM-Scl100, PM-Scl75, Jo-1, SRP, PL-7, PL-12, EJ, OJ, Ro-52 and cN-1A. The test kit contains test strips coated with parallel lines of highly purified antigens. In the first reaction step, the strips are incubated with diluted patient serum samples (1:101 in sample buffer). In the case of positive samples, the specific IgG antibodies bind to the corresponding antigen site. To detect bound antibodies, a second incubation is carried out using an enzyme-labelled anti-human IgG (enzyme conjugate) that catalyses a colour reaction. Evaluation is possible by imaging directly in the incubation trays (EUROBlotOne, Euroimmun, Padova, Italy). EUROIMMUN recommends interpreting the results on the basis of signal intensity: no signal, negative; very weak band, borderline; medium to very strong band, positive. 

Sera of patients positive for anti-cN1A with LIA were further analysed with anti-cN1A ELISA (IgG) (Euroimmun Lubeck, Germany), which provides a semiquantitative assay of antibodies of the IgG immunoglobulins against cN1A. The test kit contains microtiter strips each with eight break-off reagent wells coated with cN1A. In the first reaction step, diluted patient serum (1:101 in sample buffer) is incubated in the wells. In the case of positive samples, specific IgG antibodies bind the antigens. To detect bound antibodies, a second incubation is carried out using an enzyme-labelled anti-human IgG (enzyme conjugate) that catalyses a colour reaction. 

A semiquantitative evaluation of the results is conducted by calculating the following ratio: Extinction of control or patient sample/Extinction of the calibrator (cut-off). A ratio <1.0 is negative and ≥1.0 is positive. 

### 2.5. Muscle Biopsy

An open muscle biopsy was carried out in patients who presented with at least three of the following four conditions: muscle weakness with subacute or chronic onset; muscle pain; hyperCKemia (>2 upper limit normal values) or electromyographic evidence of myopathic changes. Muscle specimens were snap frozen in liquid-nitrogen-chilled isopentane and stored at −80 °C until use. Cryostat sections (10 μm thick) underwent histological and histoenzymic staining for the diagnostic routine (haematoxylin-eosin, Gomori modified trichrome, NADH dehydrogenase, succinic dehydrogenase, cytochrome c oxidase, succinic dehydrogenase/cytochrome c oxidase double stain, acid phosphatase, PAS and Oil Red O). Indirect immunofluorescence (IIF) or immunoperoxidase (IP) staining was carried out on 7 μm thick sections on silanized glass slides to visualise Major Histocompatibility Complex-I (MHC-I, HLA-ABC) and the terminal complex of complement (C5b-9, MAC). Photographs were taken with a Zeiss AxioPlan microscope equipped with AxioVision 5.4 software (Carl Zeiss Microscopy, NY, USA).

### 2.6. Ethics

This study was approved by the local ethics committee (Rhelabus, protocol number 22271).

## 3. Results

**Figure 1:**
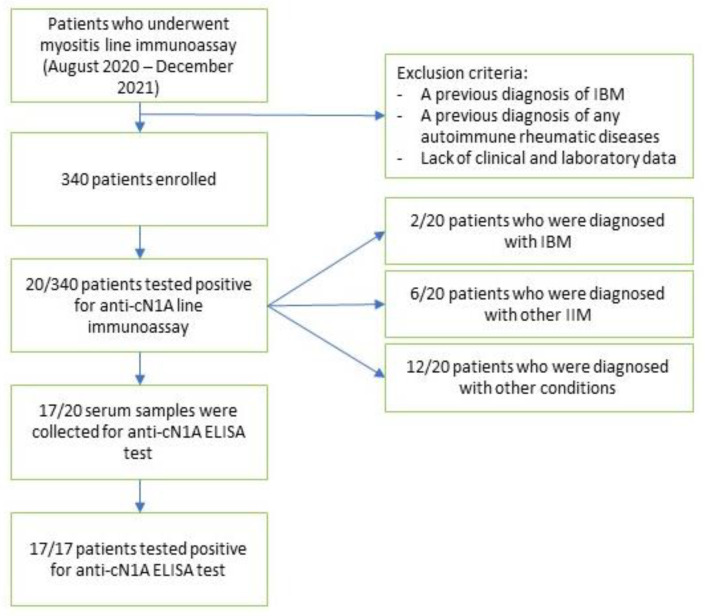
Flowchart of patient selection.

During the observation period, a total of 340 patients (Figure 1) were tested for myositis antibodies with LIA and 20 (5.88%) (15 females, 5 males, mean age 58.76 ± 18.31) tested positive for anti-cN1A. 

Notably, no patient showed concomitant positivity with LIA to anti-cN1A and any other MSA. Seventeen of the twenty samples also underwent confirmatory anti-cN1A with ELISA, and all of them tested positive (median IQR, 2.9 (1.9–4.18)). 

**Figure 2:**
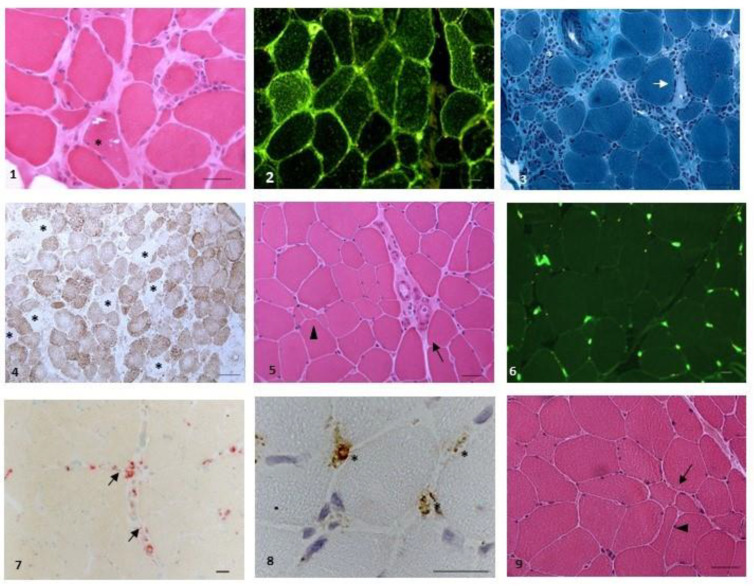
Representative pathological findings. Figures (**1**–**4**): IBM ((**1**): rimmed vacuoles (*), patient 19, haematoxylin-eosin (HE); (**2**): sarcolemmal and sarcoplasmic expression of HLA-ABC, patient 19 (IIF); (**3**): necrosis (arrow) and massive endomysial inflammation (*), patient 4, Gomori Trichrome; (**4**): large occurrence of cytochrome c (CCO) negative fibres (*), patient 4, CCO). Figures (**5**,**6**): Scleroderma associated myopathy, patient 1 ((**5**): myopathic changes, with atrophic fibres (arrowhead) and perimysial fibrosis (arrow), HE; (**6**) no upregulation of HLA-ABC, with normal localisation on endomysial small vessels and no localisation on fibres (IIF)). Figure (**7**): myositis with microangiopathy, patient 13 (small perivascular inflammatory deposits reactive for acid phosphatase). Figure (**8**): myositis DM-like, patient 9 (deposits of C5b-9 on small endomysial vessels (*), IP). Figure (**9**): seronegative arthritis (aspecific myopathic changes, with increased variation of fibre diameters (arrow) and angulated fibres (arrowhead), HE). Scale bar was set at 20 mm.

Anti-nuclear antibodies (ANA) were positive in eight out of twenty patients (titre ranging from 1:160 to 1:640). Muscle biopsy (Figure 2) was performed in ten out of twenty (50%) patients. The clinical, serological and histological (Figure 2) features of the cohort of anti-cN1A-positive patients are summarised in Table 1. 

### Clinical Profile of the Cohort

Of the twenty patients who tested positive for anti-cN1A with LIA, two were diagnosed with IBM on the basis of clinical and muscle biopsy findings. Three patients were diagnosed with dermatomyositis (DM), two with polymyositis (PM), one with scleromyositis and all underwent muscle biopsy; two of them also underwent a magnetic resonance imaging (MRI) of the thighs and a Power Doppler US of the muscle. All three patients with DM showed concomitant skin involvement, two also suffered from arthritis and one from dysphagia. Lung involvement, which was evaluated using lung function tests and high-resolution computed tomography, was excluded in all of them. The patient with scleromyositis also suffered from scleroderma, Raynaud phenomenon, nailfold videocapillaroscopy abnormalities, peripheral neuropathy and heart involvement (myocarditis and myocardial fibrosis). 

The outcome was favourable in five of the six patients who had IIM other than IBM, who were variously treated with oral glucocorticoids, Methotrexate, Mycophenolate mofetil and intravenous immunoglobulins, while the sixth patient was lost to follow-up.

Among the patients not suspected to have IIM, six were diagnosed with seronegative arthritis, two with undifferentiated connective tissue disease, one with myasthenia gravis, one with hypersensitivity pneumonia, one with metastatic pancreas carcinoma, while in one no definite diagnosis was reached.

Five of the six patients suffering from seronegative arthritis had sacroiliitis, which was detected using MRI and fulfilled ASAS criteria for spondylarthritis. One of them also had relapsing panuveitis, which did not respond to topical steroids or to two anti-TNFα agents. The last patient was diagnosed with elderly onset rheumatoid arthritis. In order to exclude concomitant myositis, one patient with spondylarthritis also underwent a contrast-enhanced MRI of the thighs and arms, which failed to detect oedema.

One of the two patients with undifferentiated connective tissue disease also suffered from bilateral symmetric polyarthritis, which responded well to low doses of glucocorticoids and hydroxychloroquine.

The patient with hypersensitivity pneumonia had a rapidly progressive form of interstitial lung disease and eventually underwent lung transplant due to the onset of respiratory failure despite concomitant treatment with Nintedanib. No definite aetiology of hypersensitivity pneumonia was found.

## 4. Discussion

The performance of the anti-cN1A antibody as a diagnostic biomarker for IBM is not yet fully defined. To the best of our knowledge, this is the first study with LIA and ELISA on a large cohort of anti-cN1A-positive patients without a previous diagnosis of IBM. Contrary to data in the literature, our study showed that anti-cN1A has low specificity in the diagnosis of IBM, which was confirmed in two out of twenty (10%) anti-cN1A-positive patients. Five of them eventually showed clinical and/or histological findings consistent with IIM (DM and PM), while only one did not meet any precise diagnosis for an autoimmune disease. 

In a previous study [20], a dot blot assay, using a moderate reactivity cut-off, was applied to 200 patients (47 with IBM) and showed 70% sensitivity and 92% specificity [20]. Likewise, an immunoprecipitation assay applied to 266 patients (94 with IBM) showed 60% sensitivity and 91% specificity.

Further studies demonstrated a high reactivity of cN1A autoantibodies in 33–34% of IBM patients against low cross-reactivity in the control groups (4–5% PM, 0–4% DM, 0–3% neuromuscular disorders and 0% in healthy controls) [4,21]. The prevalence of cN1A autoantibodies was recently investigated in other autoimmune diseases. They were detected in up to 36% of patients with Sjögren’s syndrome and up to 20% of patients with systemic lupus erythematosus, decreasing the overall specificity [6]. Although the analysis of anti-cN1A-positive versus anti-cN1A-negative IBM patients showed no correlation with age, duration of symptoms, weakness, antinuclear autoantibodies or MSA or MAA status, the rarity of anti-cN1A in PM and DM patients led to the consideration of autoantibodies as a key marker for differentiating myositis subtypes.

Accordingly, a positive test result in a patient with a muscle disease should be considered highly predictive of IBM. A negative test does not rule out IBM. The prevalence of anti-cN1A antibodies in non-neuromuscular autoimmune diseases is more controversial and may affect the diagnostic specificity of these assays for IBM [6]. 

Our findings do not confirm that anti-cN1A has a good diagnostic accuracy for IBM. The high incidence of autoimmune diseases in patients carrying anti-cN1A demands an accurate and cautious diagnostic work-up. Indeed, only one patient in our cohort was not diagnosed and three were eventually diagnosed with non-inflammatory conditions. This indicates that anti-cN1A positivity, just like positivity for other antibodies such as NOR90 [7] and anti-DSF-70 [22]—while not specific—should suggest that the patient be assessed for a concomitant rheumatic and/or autoimmune disorder. In particular, we found a percentage of cases of DM and PM way higher than reported in the literature among our anti-cN1A-positive patients (27.27% vs. 0–4%) [21,23]. It would be of interest to study whether anti-cN1A positivity in PM and DM is associated with definite clinical features as well as a better or worse prognosis and response to treatment.

It should be remarked that in our cohort, MSA and anti-cN1A were mutually exclusive: patients positive for anti-cN1A were never positive for any other MSA or MAA, whereas some were positive for other autoantibodies unrelated to any rheumatic disorder.

Our study compared LIA and ELISA methods in the assessment of anti-cN1A antibodies, finding excellent concordance between them. All patients who tested positive with ELISA were also positive with LIA. This result confirmed the diagnostic reliability of LIA for myositis.

A limitation of our study was that its monocentric design and retrospective nature did not allow us to draw any firm conclusion. Secondly, our cohort only included patients with symptoms suggesting muscular or rheumatic disorders, and therefore, did not evaluate the prevalence of anti-cN1A in healthy subjects, which indeed was not one of our aims. Likewise, the relatively high prevalence of IIM in our cohort was because our patients had muscle symptoms investigated using myositis line immunoassay. A third limit was the brief observation period, which prevented us from evaluating the prognosis and long-term outcome of our anti-cN1A-positive patients. Fourth, due to technical reasons, only 17 out of 20 patients underwent a confirmatory ELISA test. Finally, due to the design of our study, in which we included only seropositive patients, we were not able to assess the sensitivity of anti-cN1A for IBM.

## 5. Conclusions

Our study suggests excellent concordance between LIA and ELISA for detecting anti-cN1A. LIA may be a rapid and useful adjunct and could replace ELISA for this purpose. Anti-cN1A is far from being confirmed as a specific biomarker for IBM. Nevertheless, the many cases of autoimmune diseases, mainly muscular, in our cohort suggest that patients positive for anti-cN1A should be assessed with caution and should undergo a muscle biopsy.

## 6. Key Messages

- An excellent concordance was achieved between line immunoassay and ELISA test for anti-cN1A antibodies
- anti-cN1A appears far from being considered a specific biomarker for inclusion body myositis (IBM)
- patients carrying anti-cN1A positivity warrant a cautious assessment, inclusive of muscle biopsy

## Figures and Tables

**Table 1:** Clinical, serological and histological features of the patients. List of abbreviations: AMA-M2 (pyruvate–dehydrogenase complex); M2-3E (fusion protein of the E2 subunits of the alpha-2-oxoacid dehydrogenase); anti-SM: anti-striated muscle; anti-Tg: anti-thyroglobulin; anti-TPO: anti-thyroperoxidase; DM: dermatomyositis; IBM: inclusion body myositis; PM: polymyositis; SpA: spondyloarthritis; UCTD: undifferentiated connective tissue disease.

n	Age	Gender	Definite Diagnosis	Other Autoantibodies	ANA Titre	ANA Pattern	ELISA Anti-cN1A Ratio (v.n. < 1.0)	Biopsy Findings
1	68	F	Scleromyositis	anti-CENP, AMA-M2, M2-3E	1:160	AC-3	3.86	Myopathic changes, no inflammation
2	30	M	SpA	-	-	-	1.9	-
3	79	F	Seronegative arthritis, myopathic changes	-	1:320	AC-1	5.3	Mild myopathic changes, no inflammation
4	69	F	IBM	-	-	-	5.21	Rimmed vacuoles, inflammation, mitochondrial changes
5	23	M	SpA	-	-	-	1.49	-
6	64	F	UCTD	anti-RNA-POL III	1:160	AC-8	2.9	
7	66	F	PM	anti-Tg, anti-TPO	1:640	AC-4	2.55	Myopathic changes. Endomysial inflammation, necrosis and regeneration
8	53	F	Seronegative arthritis, panuveitis	anti-Tg, anti-TPO	1:160	AC-18	1.01	-
9	84	M	DM	-	-	-	5.79	Microvasculopathy, myopathic changes
10	49	F	Seronegative arthritis	-	-	-	3.61	
11	80	F	DM	-	1:160	AC-1	4.17	Mild myopathic changes, microvasculopathy
12	62	F	Myasthenia gravis	anti-SM	1:320	AC-1	Not performed	
13	67	F	DM	-	-	-	4.28	Mild perivascular inflammation
14	40	F	SpA	-	1:320	AC-15	1.77	
15	50	F	PM	-	1:160	AC-4	1.6	Mild aspecific myopathic changes
16	85	F	Pancreatic ductal adenocarcinoma	-	-	-	2.86	
17	55	F	Hypersensitivity pneumonia	-	-	-	2.71	
18	41	F	No definite diagnosis-	-	-	-	4.18	
19	86	M	IBM	-	-	-	Not performed	Rimmed vacuoles, inflammation, mitochondrial changes
20	51	F	UCTD	-	1:320	AC-1	Not performed	Myopathy, no inflammation

## Data Availability

The data presented in this study are available on request from the corresponding author.

## Abbreviations

cN1A: cytosolic 5′-nucleotidase 1A; IBM: inclusion body myositis; ELISA: enzyme-linked immunoassay; IIM: idiopathic inflammatory myopathies; Ig-: immunoglobulin-; DM: dermatomyositis; PM: polymyositis; EMG: electromyography; GCs: glucocorticoids; ANA: Anti-nuclear antibodies.
